# Wild Type p53 Transcriptionally Represses the SALL2 Transcription Factor under Genotoxic Stress

**DOI:** 10.1371/journal.pone.0073817

**Published:** 2013-09-06

**Authors:** Carlos Farkas, Carla P. Martins, David Escobar, Matias I. Hepp, David B. Donner, Ariel F. Castro, Gerard Evan, José L. Gutiérrez, Robert Warren, Roxana Pincheira

**Affiliations:** 1 Departamento de Bioquímica y Biología Molecular, Facultad de Ciencias Biológicas, Universidad de Concepción, Concepción, Chile; 2 Helen Diller Family Comprehensive Cancer Center, University of California San Francisco, San Francisco, California, United States of America; Weizmann Institute of Science, Israel

## Abstract

SALL2- a member of the Spalt gene family- is a poorly characterized transcription factor found deregulated in various cancers, which suggests it plays a role in the disease. We previously identified SALL2 as a novel interacting protein of neurotrophin receptors and showed that it plays a role in neuronal function, which does not necessarily explain why or how SALL2 is deregulated in cancer. Previous evidences indicate that SALL2 gene is regulated by the WT1 and AP4 transcription factors. Here, we identified SALL2 as a novel downstream target of the p53 tumor suppressor protein. Bioinformatic analysis of the SALL2 gene revealed several putative p53 half sites along the promoter region. Either overexpression of wild-type p53 or induction of the endogenous p53 by the genotoxic agent doxorubicin repressed SALL2 promoter activity in various cell lines. However R175H, R249S, and R248W p53 mutants, frequently found in the tumors of cancer patients, were unable to repress SALL2 promoter activity, suggesting that p53 specific binding to DNA is important for the regulation of SALL2. Electrophoretic mobility shift assay demonstrated binding of p53 to one of the identified p53 half sites in the Sall2 promoter, and chromatin immunoprecipitation analysis confirmed *in vivo* interaction of p53 with the promoter region of Sall2 containing this half site. Importantly, by using a p53ER^*TAM*^ knockin model expressing a variant of p53 that is completely dependent on 4-hydroxy-tamoxifen for its activity, we show that p53 activation diminished SALL2 RNA and protein levels during genotoxic cellular stress in primary mouse embryo fibroblasts (MEFs) and radiosensitive tissues *in vivo*. Thus, our finding indicates that p53 represses SALL2 expression in a context-specific manner, adding knowledge to the understanding of SALL2 gene regulation, and to a potential mechanism for its deregulation in cancer.

## Introduction

SALL2 is a member of the Spalt (Sal) family of transcription factors conserved from *C. elegans* to humans. Human SALL genes, SALL1, SALL3 and SALL4 were shown to be involved in normal development, and implicated in several genetic disorders, specifically congenital syndromes affecting limb, ear, kidney, and heart development. However, unlike SALL1, SALL3 and SALL4, no human congenital malformation to date has been associated with SALL2 deficiency [1,2].

We previously identified SALL2 as a novel interacting protein of neurotrophin receptors and showed that it plays a role in neuronal function [3]. Bohm et al reported that Sall2 knockout mice present strain-dependent neural tube defects (NTD) [4]. In agreement with this and our study, a decrease on SALL2 expression has been linked to neural tube defects induced by valproic acid [5].

Besides its role in neuronal development and neurogenesis, several evidences suggest that SALL2 plays a role in cancer. In support of a tumor suppressor function, SALL2 maps to a chromosomal region related to haploinsufficiency in some ovarian carcinomas [6]. Mouse Sall2 was identified as a target of polyoma virus l large T antigen. Sall2 inhibited viral DNA synthesis, and the binding of Sall2 by large T overcame this inhibition, suggesting that Sall2 negatively regulates cell growth in some contexts [7]. Li et al subsequently reported that stable overexpression of SALL2 reduced the tumorigenicity of an ovarian cancer cell line in nude mice, accompanied by increased expression of p21^WAF^ (p21), a cell cycle inhibitory protein [8]. In agreement with the effect on p21, we found that SALL2 increases p21 promoter activity and p21 protein expression in neuronal cells [3]. Consistent with a negative effect on cell proliferation, SALL2 was involved in entry into cellular quiescence in human fibroblasts [9].

According to all of the above studies, SALL2 is expected to act as a tumor suppressor. Contradictory, SALL2 is found upregulated in several cancers. Microarray studies identified SALL2 as one of the 27 signature-genes highly expressed in Wilm’s tumor [10]. Furthermore, cDNA expression profiling on soft tissue tumors identified SALL2 in a group of genes characteristically expressed by synovial sarcomas [11]. SALL2 was also identified among the overexpressed genes in squamous cell carcinoma (SCC) of the tongue, and was proposed as a prognosticator of head and neck SCC since its expression is associated with depth of invasion and advance T stage [12,13]. Lately, a transcriptome study indicated that SALL2 is upregulated in Testicular Germ Cell tumor (TGC) and may be involved in TGC tumorigenesis [14]. In addition, SALL2 has been implicated as a predictor of lymph node metastasis in breast cancer [15]. Since SALL2 is a transcription factor, deregulation of its expression should affect gene expression, thereby explaining its association with cancer. However, the molecular mechanisms that regulate its activity and expression are still unclear.

Understanding how SALL2 is regulated under different cell scenarios should help to understand its deregulation in cancer. Between the pathways that regulate Sall genes, decapentaplegic (*Dpp*), an orthologue of transforming growth factor-β (TGFβ) in *Drosophila* , acts as an upstream regulator of spalt [16]. In fish, Medaka*Spalt* regulation has also been linked to hedgehog (Hh) activity [17], and Nerve Growth Factor (NGF) was shown to positively regulate SALL2 in rat neurons [3]. However, less is known about SALL2 transcriptional regulation. The SALL2 gene contains two alternative promoters, named P1 and P2 that could result in two proteins that differ in 25 amino acids [18]. Both promoters contain putative sites for the Wilms tumor-1 protein (WT-1), and are repressed by WT-1 [19]. Recently, it was shown that the Activator Protein 4 (AP4) transcription factor positively regulates SALL2 in serum-deprived fibroblasts, an effect that was negatively regulated by TGFβ by inhibiting transcription of both SALL2 and AP4 in these cells [20]. Latest evidence indicates that the P2 promoter of SALL2 is CpG-rich and susceptible to silencing by methylation, a modification confirmed in OVCA-derived cell lines and in the majority of primary tumors, supporting a role of SALL2 as a suppressor of ovarian cancers [21].

We now identified SALL2 as a novel downstream target of the p53 tumor suppressor protein, which is frequently inactivated in cancer. We show that p53 binds to the SALL2 P2 promoter gene, and that SALL2 expression is negatively regulated by p53. The effect of p53 on SALL2 levels was dependent on P53 transcriptional activity. Importantly, by using a p53ERTAM knockin (*p53* ^ER/ER^) mouse model, that can be reversibly and rapidly toggled between p53 wild-type (wt) and knockout states [22], we demonstrated that p53 represses SALL2 levels during genotoxic stress in primary mouse embryo fibroblasts (MEFs) and radiosensitive tissues *in vivo*. Our data indicate that SALL2 transcription factor is regulated by p53 under DNA damage, indicating a potential mechanism for SALL2 deregulation in cancer.

## Materials and Methods

### Reagents

Doxorubicin and 4-Hydroxy-Tamoxifen were purchased from SIGMA (St. Louis, MO, USA). The p21 monoclonal (H5) and p53 monoclonal (DO1) antibodies were obtained from Santa Cruz Biotechnology (Santa Cruz, CA, USA). Anti-actin was obtained from SIGMA, monoclonal anti-GAPDH antibody was obtained from Biodesign (Memphis, TN, USA) and polyclonal anti-SALL2 was obtained from CeMines (Evergreen, CO, USA). Phospho p53 (Ser15) antibody was from Cell Signaling (USA) and p53 PAb421 antibody was from EMD Millipore (Darmstadt, Germany).

### Plasmids

Two truncated forms of 1.2 kb and 2.3 kb of the Sall2 P2 promoter were cloned into the pGL3 vector (Promega). The 1.2 kb SALL2 promoter fragment was amplified from genomic DNA of HEK293T cells by PCR with TaKaRa, LA taq (Clontech), cloned into TOPO TA vector (Invitrogen), and then subcloned between the XhoI and HindIII sites of the pGL3 vector. To generate the 2.3 kb fragment, a 1.1 kb fragment upstream of the 1.2 fragment was amplified by PCR and then linked to the 5’ end of the 1.2 kb SALL2 promoter fragment between the KpnI and XhoI sites of the pGL3 vector. Primers used for PCR reactions and corresponding products size are summarized in Table S1. Fidelity of the promoter fragment sequences were confirmed by dideoxynucleotide sequencing using GL2 and RV3 primers. P53 wild type and p53 mutant plasmids were kindly provided by Dr. Bert Vogelstein (Johns Hopkins Medicine, U.S.A). The P2 SALL2 core promoter (344 pb) pGL3 construct was kindly provided by Dr. Thomas Benjamin (Dana Farber, Harvard Cancer Center, USA), and was previously reported [20].

### Cell culture

HEK 293T human kidney epithelial cells (ATCC^®^ CRL-11269^™^), H1299 p53-null human nonsmall lung carcinoma (ATCC^®^ CRL-5803^™^), p53 wild type (+/+) and null-derivatives (-/-) HCT116 colon cancer cells kindly provided by Dr. B. Vogelstein (Johns Hopkins) [23], and p53 ^ER/ER^ MEFs isolated from the p53^ER/ER^ embryos were cultured in DMEM (Hyclone) supplemented with 10% (v/v) Fetal Bovine Serum (FBS, Hyclone), 1% glutamine (Invitrogen), and 1% penicillin/streptomycin (Invitrogen). Rat PC12 pheochromocytoma cells (ATCC^®^ CRL-1721^™^) were cultured in DMEM supplemented with 10% (v/v) horse serum, 5% (v/v) FBS, 1% glutamine, and 1% penicillin/streptomycin. Experiments with p53 ^ER/ER^ MEFs were performed with early passages (prior to passage 6). When indicated, we added 100 nM of 4-hydroxytamoxifen (Sigma) in 100% ethanol, or an equal volume of ethanol control, to the medium. The cells were passaged using a standard 3T9 protocol. For genotoxic stress and p53 activation, doxorubicin and etoposide were added to the cell culture at indicated concentrations and times (see figure legends).

### Isolation of primary MEFs and genotyping

Mice were group housed under standard conditions with food and water available ad libitum, and were maintained on a 12-h light/dark cycle. Mice were fed a standard chow diet (Lab Diet, ProLab) containing no less than 5% crude fat and were treated humanely in compliance with the US National Institutes of Health guidelines for animal care and use. Mice studies were reviewed and approved by the UCSF laboratory animal research center (AN078648-02) and the Animal Ethics Committee of the Chile’s National Commission for Scientific and Technological Research (CONICYT, Protocol FONDECYT project 1110821)

Fibroblasts from p53 ^ER/ER^ mice were prepared from embryos at 12.5-13.5 days postcoitum as previously described [22] and cultured as described above. For mice genotyping, a piece of mouse tail was incubated at 55°C for 16h in 300 µL lysis buffer containing Tris 100mM, EDTA 5mM, SDS 0.2%, NaCl 200mM and 60 µg proteinase K. One microliter of genomic DNA was used for PCR analysis. p53 PCR was carried out with the following oligonucleotides: (forward; CCTCCAGCCTAGAGCCTTCCAAGC), (reverse; GGTGAGATTTCATTGTAGGTGCC), and (Neo; GCACACAAACTCTTCACCCTGC). The sizes of the PCR products are 430 bp for the WT and 700 bp for the null mutant. PCRs were performed for 32 cycles with annealing temperature for p53 at 66°C.

### Western blot analysis

Cell lysates (25-50 µg of total protein) were subjected to SDS-PAGE, transferred to PVDF membrane (Immobilon, Millipore) and then analyzed by Western blot, following standard procedures.

### Induction of DNA damage on p53 ^ER/ER^ mice

For experiments with p53 ^*ER/ER*^ mice, we followed the previously described method [22]. Briefly, to restore p53 to wild-type status in 8 weeks old p53^*ER/ER*^ mice, we administered by intraperitoneal injection 100 µl of 10 mg/ml tamoxifen (SIGMA, T5648) in peanut oil carrier at the indicated frequencies. Control mice were given peanut oil carrier alone. Then mice were treated with 5 Gy of ionizing radiation from a Cs137 source and tissues were collected for RNA and protein analysis.

### RT-PCR

Total RNAs were extracted from cells with Trizol reagent (Life Technologies, Inc.) according to the manufacturer’s instructions. Before RT-PCR, the RNA was treated with Turbo DNase (Ambion) to eliminate any residual DNA from the preparation. For RT–PCR, the RNA (1.0 µg) was reverse transcribed into cDNA with random hexamer using the ImProm-II Reverse transcription System (Promega). Expression of gene was determined by PCR. The conditions for SALL2 were 95°C 30s, 55°C 45s and 72°C 45s for 32 cycles. For cyclofilin and for P21 were 95°C 30s, 55°C 45s and 72°C 45s for 30 cycles and 26 cycles respectively. The primers were 5’- CGGATACCCATTGTGTCCTA (forward) and 5’ -CAGCATTTGGCACAGACTTG (reverse) for **Sall2**, and 5’ - TGGAGATGAATCTGTAGGACGAG (forward) and 5’ - TACCACATCCATGCCCTCTAGAA (reverse) for **cyclofilin.**

### Transient Transfections and Reporter Gene Assays

***Toevaluatetheeffectofp53ontheSall2P2promoter***, 293T cells or *p53-*null MEFs were transfected with 1.0 µg of control or p53 plasmid, 1.0 µg of SALL2 P2 promoter (2.3kb, 1.2 kb or 344bp) and 0.5 µg of β-Galactosidase (β-Gal). H1299 cells were transfected with 0.25, 0.5 or 1.0 µg of control or p53 plasmid, 1.0 µg of SALL2 P2 promoter and 0.5 µg of β-Gal. After 48 h, the transfected cells were washed with phosphate-buffered saline, lysed with reporter assay lysis buffer (Promega), and spun at 10,000 × *g* to pellet cell debris. The supernatant was then assayed for luciferase and β-Gal activity using the manufacturer’s suggested protocols (Promega). Luminescent reporter activity was measured using a Luminometer (Victor3, PerkinElmer). All transfections were normalized to β-Gal activity and performed three times in triplicate. Luciferase values are expressed as relative luciferase unit (R.L.U). Statistical significance of X *versus Y* -treated samples was determined by one-tailed Student’s *t* test.

***Toevaluatetheeffectofendogenousp53activationontheSALL2P2promoter***, HCT116 (p5*3*wt) cells were transfected with 1.0 µg SALL2 P2 promoter and 0.5 µg β-Gal. Twenty-four hours after transfection cells were treated with 1.0 µM doxorubicin for 12 and 24 h. Finally, cells were washed, lysed and then assayed for luciferase and β-galactosidase activity as above.

### EMSA and Chromatin Immunoprecipitation Assays

***ElectrophoreticMobilityShiftAssays(EMSA)*** were performed using nuclear extracts prepared according to the Dignam method [24]. These extracts were obtained from HCT116 cells (p53WT, p53-null and p53-null cells transiently transfected with a vector coding for wild-type p53). The presence of p53 in these extracts was confirmed by Western blot. ^32^P-end-labeled oligonucleotides were used in these assays (See sequences in Table S2). Binding reactions (20 µL) were performed using 20 fmol of each labeled probe and 1.0-1.5 µg salmon sperm DNA (Invitrogen, 15632-011) or poly(dI-dC) (Roche, 10108812001). Where indicated, 8.0 µg of nuclear extract and 100 ng of PAb421 antibody (EMD Millipore) were added. Binding reactions were adjusted to the following final conditions: 20mM HEPES-KOH pH 7.9, 100mM KCl, 1mM EDTA, 0.05% Nonidet P40, 0.5mM PMSF, 3mM DTT, 10% v/v glycerol, 50 mg/ml BSA. After a 30 minutes incubation at 30^°^C the samples were subjected to electrophoresis in a non-denaturing polyacrylamide gel (4% w/v; acrylamide: bis-acrylamide ratio 40:1; 0.3X TBE). Afterwards, gels were dried and subjected to autoradiography.

***Chromatinimmunoprecipitation*** was carried out as described previously [25] with the following modifications: HCT116 cells were grown on 100-mm dishes to 80% confluency and then treated with 1 µM of doxorubicin for 12 or 24 hours. Cell nuclei were sonicated to shear DNA in 300 µl of sonication buffer, using a Sonic Vibracell VCX 130 apparatus (8 times, 15s on/20s off each time, 100W potency), obtaining lengths between 300 and 600 bp. Immunoprecipitations were carried out overnight at 4 °C using 3 µg p53 (DO-1, Santa Cruz) or normal mouse IgG antibodies (sc- 2015, Santa Cruz) and 40 µg of chromatin. DNA was analyzed by conventional PCR directed to specific regions of SALL2 P2 proximal promoter and P21 promoter (a positive control for p53 ChIP), using the primers summarized in Table S1. All PCR reactions (Taq polymerase, Invitrogen) contained 0.05% v/v DMSO and 0.05% v/v of Tween 20. Cycling conditions for all reactions were as follows: initial denaturation at 94°C for 2.5 min, then 25 cycles with 94°C for 45 seconds (denaturation), 52°C for 45 seconds (annealing) and 72°C for 30 seconds. Thirthy-two cycles were used in the case of the P21 amplicon.

## Results

### The p53 tumor suppressor inhibits SALL2 P2 promoter activity

**Figure 1:**
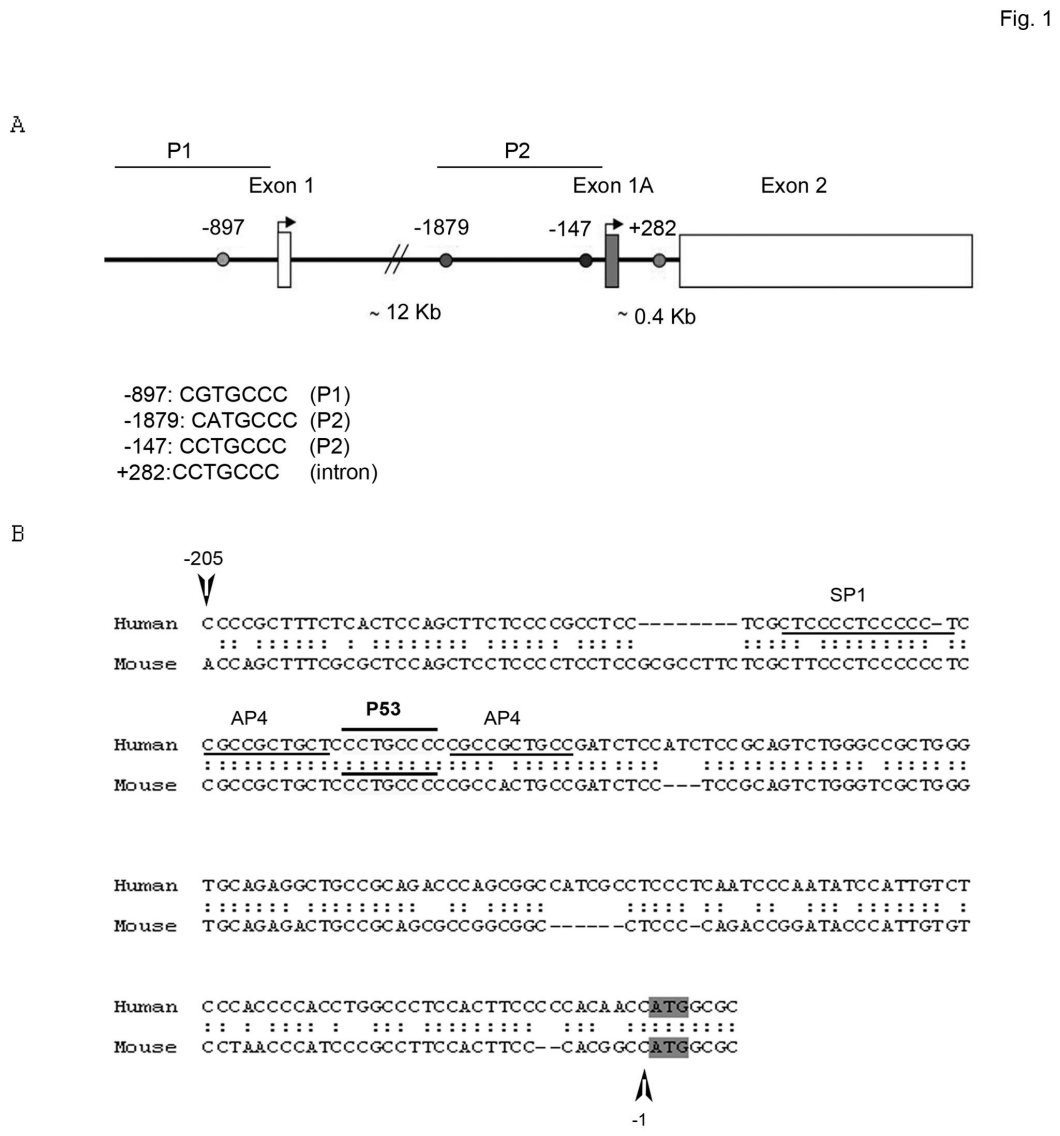
Identification of putative p53 half sites in the human SALL2 promoter gene. **A**. Schematic representation of the SALL2 gene (NM_005407) showing exon 1, exon 1A and exon 2, and the positions of putative p53 half sites identified by bioinformatic analysis of the alternative promoters P1 (-897) and P2 (-1879 and -147), and of the intron region (+282). **B**. Promoter alignment of human and mouse SALL2 promoter regions by rVISTA and LALIGN. A 210 bp fragment upstream of the ATG of human and mouse exon 1A is schematized and the conserved p53 half site location is indicated. Flanking the p53 half site, two putative activator protein 4 (AP4) sites are underlined. The location of the putative stimulating protein 1 (SP1) site is also shown. Arrows and numbers refer to the **ATG** of Exon 1A.

In an attempt to understand SALL2 deregulation in cancer, we searched for potential cancer-related transcription factor binding sites in the SALL2 gene. By using a virtual laboratory for identification of putative transcription factor binding sites in DNA sequences (ALGENN PROMO) [26,27] and a regular expression search for binding sites against a mammalian promoter database (TRED, TESS, TRANSFAC and MAPPER), we evaluated the human SALL2 gene (accession number NM_005407). Importantly, we identified four putative half sites for the p53 tumor suppressor protein in the SALL2 gene. One putative site was located in the P1 promoter (for exon 1, position -897 to the first ATG) and two putative sites were located in the P2 promoter (for exon 1A, positions -1879 and -147 to the first ATG). In addition, a putative p53 binding site was located in the intron region between exon1A and exon2 (position + 282 to the first ATG of exon 1A) (Figure 1A). Furthermore, we used p53FamTag database [28], which combines bioinformatics predictions with microarray data, to screen for p53 binding sites in the SALL2 locus. Using p53FamTag database, four sequences, unrelated to the predicted above were identified. Two potential p53 sites were located at positions -3000 and -1132 from start of exon 1, and two positions located at -4300 and -4700 from start of exon 1A (Figure S1). Finally, the human SALL2 and murine Sall2 promoter sequences were aligned for sequence comparison. Promoter alignment using rVISTA and LALIGN identified a conserved (67.5% identity) region that lays 1100bp upstream of the ATG translation site of exon 1A in the human and murine promoters. The putative p53 half site identified at position -147 bp is conserved (Figure 1B), and contains the sequence C **CT** GCCC. A “CWWG” core containing the dinucleotide core **CT** previously associated with p53 activating or repressing activity depending on the p53RE half-site background where is located [29]. All these findings suggested that p53 serves as a transcriptional regulator of the SALL2 promoters.

**Figure 2:**
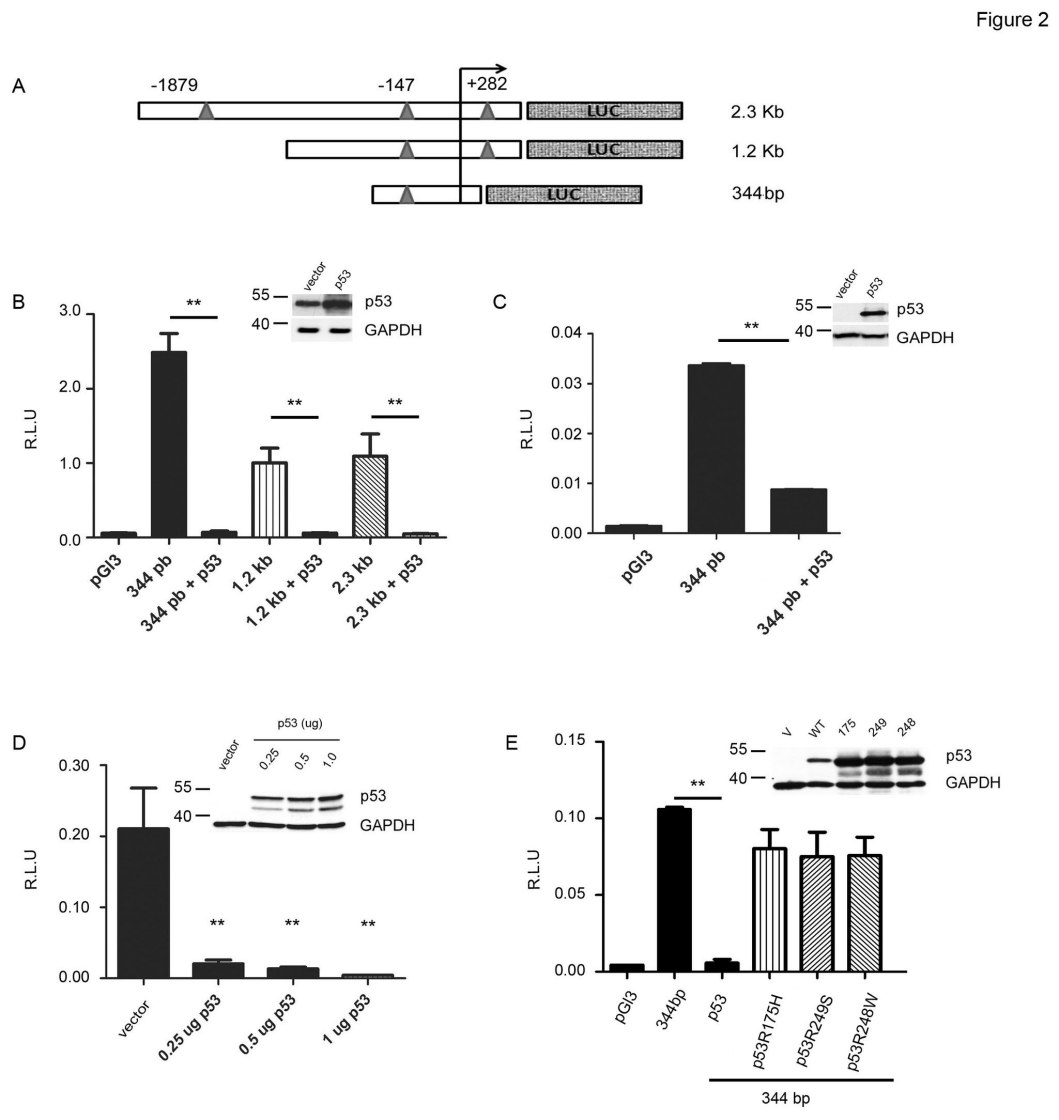
Repression of SALL2 promoter activity by wild type p53. Transient co-transfections of the SALL2 P2 promoter-luciferase reporter constructs with or without p53 into different cell lines were performed as described under “Materials and Methods”. Luciferase activity was measured from cell lysates and normalized to β-galactosidase activity, and promoter activity was expressed as relative luciferase units (R.L.U). pGL3 vector served as control. **A**. Schematic diagram of the 2.3kb, 1.2kb and 344bp fragments of the SALL2 P2 promoter-luciferase reporter constructs with triangles representing the location of p53 half sites **B**. Activity of the 344bp, 1.2kb or 2.3kb promoter constructs in the absence or presence of wild type p53 in 293T cells **C**. Promoter activity of the 344bp promoter construct in the absence or presence of p53 in p53 (-/-) Mouse embryo fibroblasts (MEFs p53 (-/-)). **D**. Promoter activity of the 344 bp promoter construct in the presence of different amounts of p53 in H1299 (p53-/-) cells. **E**. Promoter activity of the 344 bp promoter construct in the presence of wild type p53, R175H, R249S, or R248W p53 mutants in H1299 (p53-/-) cells. The results represent three independent experiments, each assayed in triplicate. Each bar represents the mean +/- standard error. Statistical significance was determined by student t-test (** p = 0.001). Western blot for p53 overexpression are shown at the top right corner of each graph, molecular weight markers are indicated on the left of the blot.

We focused our study on the SALL2 P2 promoter as it contains several putative p53 half sites, and cloned SALL2 promoter regions containing three (-1879, -147 and +282 bp) or two (-147 and +282bp) putative p53 binding sites into the pGL3 luciferase reporter vector, named here as 2.3 kb and 1.2 kb promoters, respectively. A short P2 promoter (344bp) previously described [20] was also used for analysis as it contains the p53 putative half site that is conserved in the human and mouse genes (Figure 2A). To test whether p53 regulates SALL2 gene transcription, the various SALL2 promoter luciferase reporter constructs were individually co-transfected with a p53 expression vector into 293T cells (constitutively express the simian virus 40 large T antigen, which binds to p53 and prevents it from activating transcription [30–33], but does not decrease the stability of p53 [34]), p53-null MEFs, or H1299 lung cancer cells (have a homozygous partial deletion of the TP53 gene and as a result, do not express the tumor suppressor p53 protein [35]). The activity of all SALL2 P2 promoter regions tested was dramatically repressed by the ectopic expression of p53 in the three cell lines (Figure 2B, 2C and 2D). The repressive effect of the p53-expressing vector was even observed at low concentrations of p53 (Figure 2D).

### p53 transcriptional activity represses the SALL2 P2 promoter

In order to investigate whether p53 transcriptional activity is necessary for the regulation of the SALL2 gene, we compared the effect of wild type p53 to that of transcriptional-inactive p53 mutants R175H, R249S or R248W on P2 promoter activity (344bp) in H1299 cells. In contrast to wild type p53, none of the three p53 point mutants, mutated in the DNA binding- core domain of p53, significantly affected SALL2 P2 activity (Figure 2E). Thus, these results suggested that regulation of SALL2 by p53 depends on p53 transcriptional activity.

**Figure 3:**
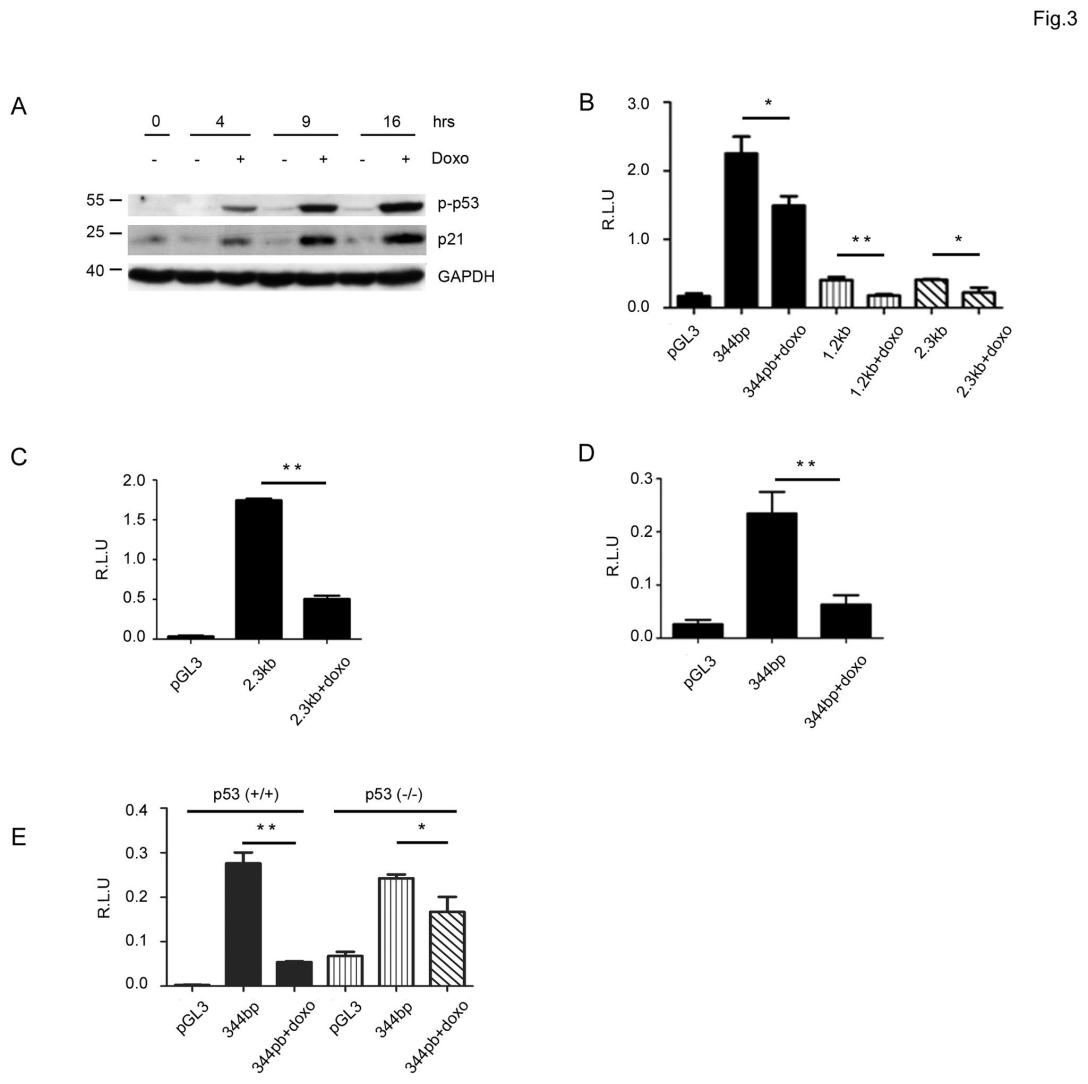
Repression of the SALL2 promoter activity by activation of endogenous p53. **A**. Human HCT116 cells (p53 +/+) were exposed to doxorubicin for 4, 9 and 16 hours, and cell lysates were used to evaluate p53 activity by western blot analysis, molecular weight markers are indicated. Levels of p21 and phosphorylated p53 are shown **B**. Transient transfections of HCT116 cells with the promoter constructs schematized in Figure 2A were performed as described under “Materials and Methods”. Twenty-four hours after transfection cells were exposed to 1µM doxorubicin or vehicle (DMSO) for 12 h and then luciferase activity was measured from cell lysates and normalized to β-galactosidase activity. Promoter activity was expressed as relative luciferase units (R.L.U). pGL3 vector served as control. The results represent at least three independent experiments, each assayed in triplicate. Each bar represents the mean +/- standard error. Statistical significance was determined by student t-test (*: p < 0.005, ** p = 0.001). **C**. Promoter activity of the 344 bp construct in HCT116 cells after 24 h of exposure to doxorubicin. **D**. Same as in C. for the 2.3 kb construct. **E**. Equal number of HCT116 (P53+/+) and (P53-/-) cells were simultaneously transfected with the 344bp construct and exposed in parallel with DMSO or doxorubicin for 24 h. Promoter activity was measured as in **B**.

To confirm that transcriptional activation of p53 is necessary for the regulation of SALL2, we tested whether activation of endogenous p53 affects SALL2 P2 promoter activity in HCT116 colon cancer cells expressing wild type p53. Cells were exposed to doxorubicin, a well-characterized activator of p53 [36], for four, nine and sixteen hours. Since phosphorylation of N-terminal serine 15 in human p53 contributes to its activation by DNA damage [37,38], we first evaluated levels of phospho-p53 (Ser15) and of its direct target p21, to confirm p53 activation by doxorubicin in these cells. Doxorubicin increased both phosphorylation of p53 and the expression of p21. The induction of p53 activity was observed after 4 hours treatment and increased over time (Figure 3A). We then evaluated the effect of p53 activation on SALL2 P2 promoter activity after 12 and 24 hours with doxorubicin. Similar to the above results with ectopic expression of p53, endogenous p53 activation in HCT116 cells decreased P2 promoter activity (Figure 3B), close to a 40% decrease after 12 hours with doxorubicin. After 24 hours treatment, we observed a further decrease (close to 76%) on transcriptional activity for both the 2.3 kb and the 344bp SALL2 P2 promoter regions (Figure 3C and D). Interestingly, we observed a significant decrease on SALL2 promoter activity in the p53-null derivatives HCT 116 cells treated with doxorubicin, suggesting some p53-independent effect of doxorubicin. However, the stronger effect of doxorubicin on HCT116 p53 wild type cells supports a role for p53 transcriptional activity in the repression of the SALL2 promoter (Figure 3E).

### P53 binds to the SALL2 promoter

**Figure 4:**
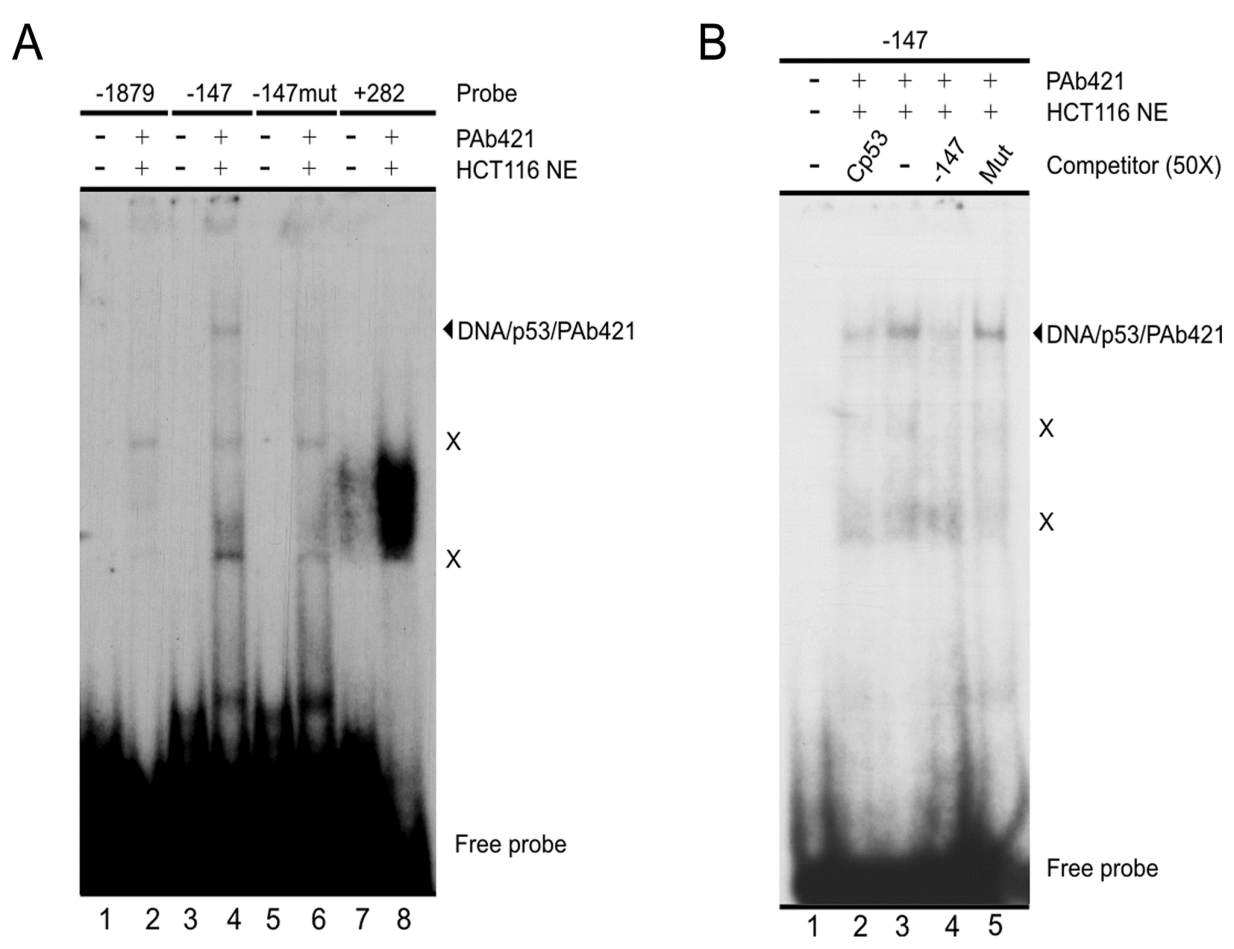
p53 binds to a DNA sequence located at -147 of the SALL2 P2 promoter. **A**. EMSA assay testing double-stranded oligonucleotide probes containing the putative p53 binding sites located at positions -1879, -147 and +282 of the SALL2 gene (see text for details). The assay includes a -147 probe with two point mutations (-147mµt). The nuclear extract used in this assay was obtained from HCT116 p53 +/+ cells. **B**. The -147 probe was used in a competition analysis, in the presence of a 50x molar excess of the unlabeled oligonucleotides Cp53, -147mµt (Mut) and -147, as indicated at the top of the figure. This assay used a nuclear extract obtained from HCT116 p53+/+ cells. **A** and **B**. The presence of nuclear extract and PAb421 antibody in the binding reactions is indicated at the top of the figures. The migration of free probe and DNA/p53/PAb421 complex is indicated at the right side of the figure, as well as migration of non-specific complexes generated in the presence of nuclear extracts (X).

Considering the observed involvement of p53 transcriptional activity in SALL2 repression and the putative p53-binding sites found in the SALL2 gene (Figure 1A), we tested the ability of p53 to bind these sites using Electrophoretic Mobility Shift Assays (EMSA). This assay was performed under conditions of p53 binding to a consensus p53-binding site (Cp53) (Figure S2). We analyzed four different double-stranded oligonucleotide probes, each containing one of the following sequences: a putative binding site located at position -1879 of the P2 promoter (-1879), putative binding site at position -147 of the P2 promoter (-147), a sequence containing two point mutations respect to the -147 probe (corresponding to nucleotides considered as key in a p53-binding site; probe named -147mµt), and putative binding site located in the intron region (+282). This assay was performed using a nuclear extract either from HCT116 p53+/+ cells (Figure 4) or HCT116 p53-null cells transfected with a vector coding for wild-type p53 (data not shown). The p53 antibody PAb421 was included in our binding reactions as p53 binding to DNA is stabilized in the presence of this antibody (Figure S2 and [39,40]). We observed p53 binding to the probe corresponding to the putative binding site located at position -147 of the P2 promoter (Figure 4A, lanes 4). A strong band with very low electrophoretic mobility was observed for the intron region +282 (Figure 4A, lane 8). Further analysis of this faster migration band indicates that was not generated by p53 binding to the probe (Figure S3). Our results indicate that the interaction of p53 with the -147 probe is specific. First, no interaction bands were detected with the -147mµt probe (Figure 4A, lane 6). Moreover, when adding a molar excess of non-labeled probes to the reactions, the Cp53 probe and the -147 probe itself, but not the -147mµt probe, efficiently competed with the interaction of p53 with the labeled -147 probe (Figure 4B).

**Figure 5:**
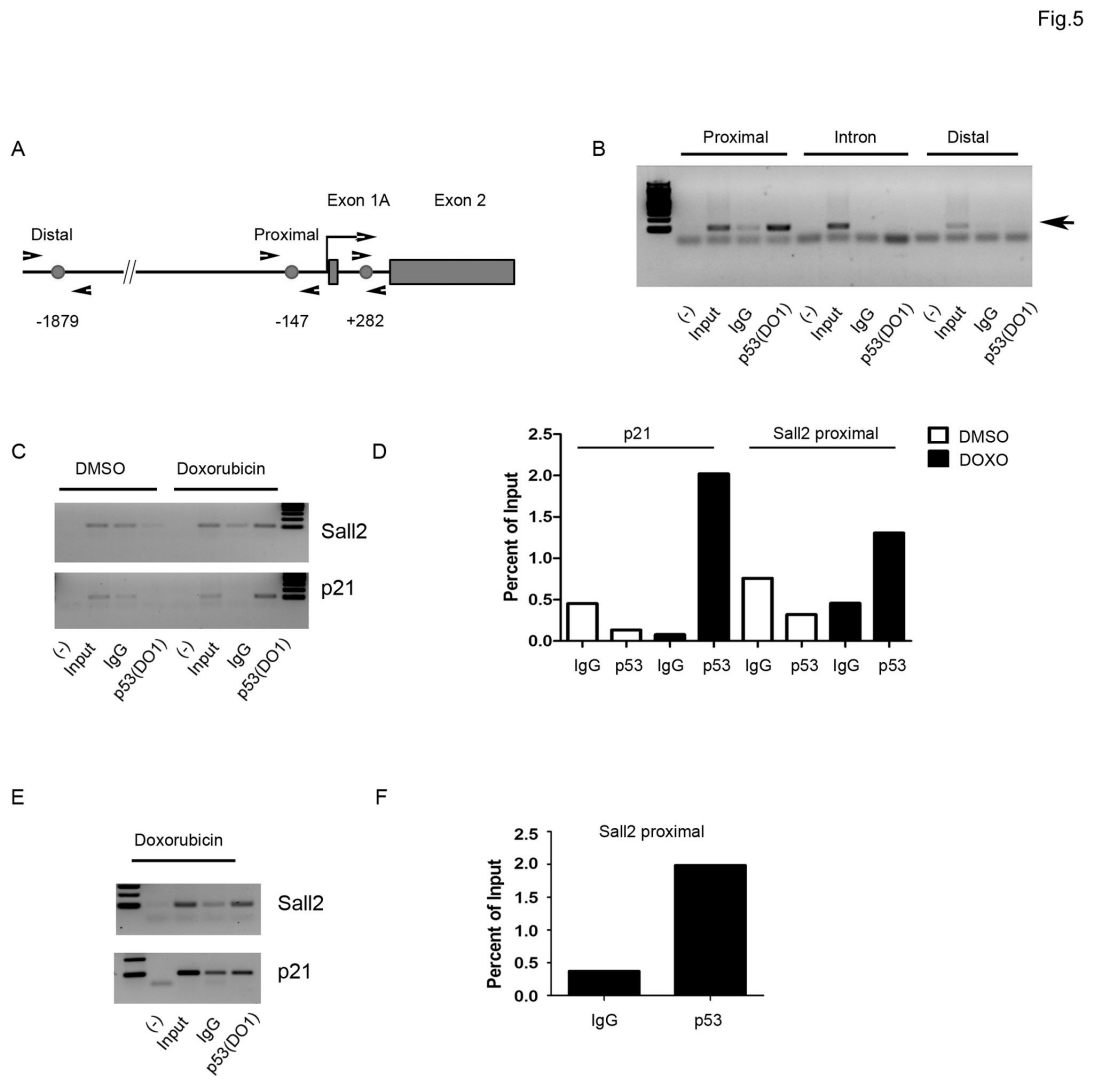
In vivo interaction of p53 with the proximal region of the SALL2 P2 promoter. **A**. Relative positions of the PCR primers for amplification of the proximal and distal regions of the SALL2 P2 promoter and the intron region are shown schematically, the arrows represent the primer set positions refer to the **ATG** of Exon 1A. The circles and numbers indicate the location of the p53 half sites described in Figure 1A. **B**. ChIP analysis for the presence of p53 on the proximal and distal regions of the SALL2 P2 promoter and the intron region after 24h treatment with doxorubicin, the arrows show the expected band size. **C**. ChIP analysis for the presence of p53 on the proximal region of Sall2 promoter after 12 h treatment with doxorubicin. The presence of p53 on the p21 promoter was used as positive control, and purified mouse IgG was used as control antibody. **D**. Densitometry analysis of a representative ChIP experiment on the p21 promoter and the proximal region of SALL2 promoter after 12 h treatment. Values are expressed as percent of input. **E**. ChIP analysis for the presence of p53 on the proximal region after 24h treatment with doxorubicin. **G**. Densitometry analysis of a representative ChIP experiment on the proximal region of SALL2 promoter after 24h with doxorubicin. All experiments were performed in triplicate.

We then performed ChIP analysis to confirm binding of p53 to the SALL2 P2 promoter in vivo. HCT116 wild-type cells were treated with doxorubicin to induce p53 activation upon DNA damage, and the p53 occupancy was examined on formaldehyde cross-linked chromatin by ChIP using a specific antibody for p53 (DO1). In parallel, PCR was conducted with input DNA, and a nonspecific IgG was used to monitor the specificity of the reaction. P53 occupancy on the P21 promoter region was used as a positive control. The analysis used a panel of 3 pairs of oligonucleotide primers that span the proximal (-147) and distal (-1879) regions of the transcription start site of SALL2 exon 1A, and the intron region (+282) between exon 1A and exon 2 (Figure 5A). Consistent with the EMSA assay, we detected p53 binding to the proximal region of exon 1A, which is conserved between the mouse and human SALL2 genes. In contrast, no binding of p53 was detected to the intron or distal region of the SALL2 gene (Figure 5B). We then tested the proximal region occupancy by p53 at 12 and 24 hours after doxorubicin treatment and observed an increase on occupancy after 24 h treatment (Figure 5E-5F) compared to that after 12 h treatment (Figure 5C and 5D). These results demonstrated that active p53 binds to the SALL2 P2 promoter in vivo, indicating a direct role for p53 in the repression of SALL2 expression.

### P53 activation diminishes SALL2 levels during genotoxic cellular stress

**Figure 6:**
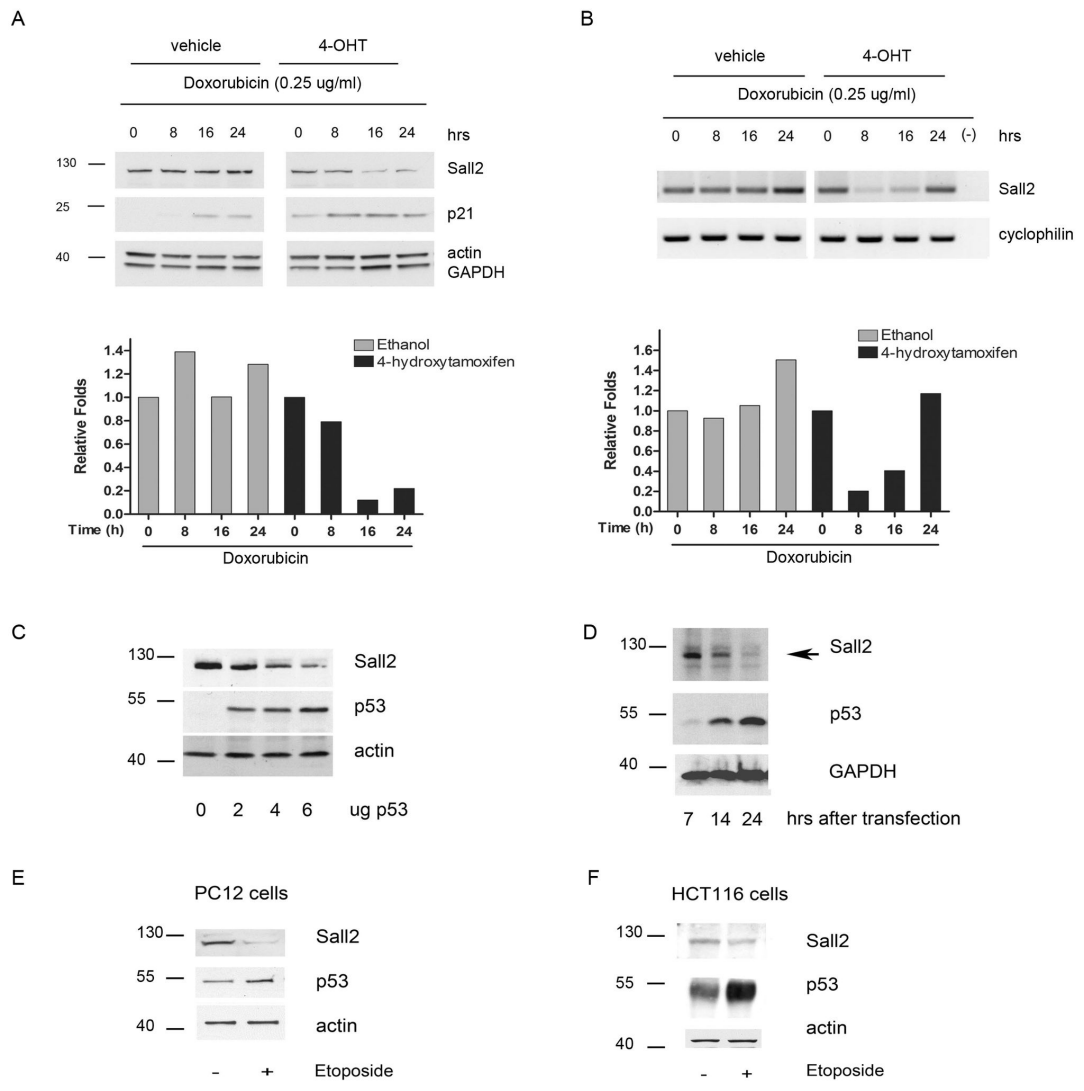
Inhibition of Sall2 expression by p53. Early passages MEFs p53^ER/ER^ were treated with 4 hydroxytamoxifen (4-OH Tamoxifen) or vehicle for 4 hours before doxorubicin treatment. **A**. Western blot analysis of SALL2 and p21, using whole-cell lysates from early-passage MEFs p53^*ER/ER*^ that were cultured in either the presence (4-OH Tamoxifen) or the absence (vehicle) of 4-hydroxytamoxifen. β-actin and GAPDH show equal loading. Shown below densitometric analysis of the data using ImageJ. Sall2 band intensities were normalized by GAPDH. Results are expressed as fold changes relative to control vehicle treated cells, and representative of three independent experiments with similar results. **B**. RT-PCR analysis of Sall2 on total RNA isolated from same experiment as in A. Cyclofilin is used as normalizing gene. Shown below densitometric analysis of Sall2 mRNA levels normalized to cyclofilin. Results are expressed as fold changes relative to control vehicle treated cells. **C**. Western blot analysis of SALL2 and p53 using total protein lysates obtained from Rat PC12 pheocromocitoma cells transfected with various concentrations of p53. Lysates were subjected to SDS-PAGE and levels of endogenous SALL2 and exogenous p53 were evaluated by western blotting. Actin shows equal loading. **D**. Rat PC12 pheocromocitoma cells were transfected with 4 µg of wild type p53 and lysates were collected after 7, 14, and 24h post transfection. Endogenous SALL2 and exogenous p53 levels were evaluated by western blotting. GAPDH shows equal loading. **E**. Western blot analysis of endogenous SALL2 and p53 in rat PC12 cells treated with 10µM Etoposide for 12h. Actin shows equal loading. **F**. Western blot analysis of endogenous SALL2 and p53 in human HCT116 (p53+/+) colon cancer cells treated with 10µM etoposide for 24h. Actin shows equal loading. For all western blot molecular weight markers are indicated on the left side.

The above-described results on the regulation of the SALL2 promoter by p53 indicated that p53 activation should decrease SALL2 expression. To demonstrate the regulation of SALL2 expression by p53 we used a well- characterized p53ERTAM knockin (p53 ^ER/ER^) mouse model [22]. In the switchable p53ERTAM knockin (p53 ^ER/ER^) mouse model [22] both copies of the endogenous p53 gene have been modified to encode a 4-hydroxytamoxifen (4- OHT) - dependent p53ERTAM protein, a fusion between p53 and a modified form of the estrogen receptor [41]. p53^ER/ER^ mice can be reversibly and rapidly toggled between p53 wild-type (wt) and knockout states by, respectively, administration or withdrawal of 4-OHT [22]. Importantly, provision of 4-OHT to either p53^ER/ER^ cells *in vitro* or tissues of p53^ER/ER^ mice *in vivo* does not itself activate p53ERTAM, but rather renders p53ERTAM competent to become activated should appropriate signals arise in such cells. Furthermore, by all tested criteria 4-OHT- ligated p53ERTAM is functionally equivalent to wt p53 [42]. We cultured early passage (passage 3 or 4) MEFs isolated from p53 ^ER/ER^ embryos in either the presence or the absence of 4-OHT and then exposed them to doxorubicin. We then assayed RNA and protein expression of SALL2 in each MEF population by RT-PCR and western blot analysis, respectively. In the presence of 4-hydroxytamoxifen, when p53 is functional, doxorubicin decreased SALL2 expression at RNA and protein levels, although they follow slightly different temporal dynamics. In the absence of 4-hydroxytamoxifen, no major changes on SALL2 protein levels were observed, except for a slight increase after 24h with doxorubicin that correlated with an increase of the mRNA (Figure 6A and 6B). Consistent with the activation of p53, doxorubicin induced expression of p21. Induction of p21 by doxorubicin was greatly reduced in vehicle treated cells compared to those treated with 4-hydroxytamoxifen. However, a slight increase in the amount of p21 was observed in the absence of 4-hydroxytamoxifen, perhaps reﬂecting some residual p53 activity or the induction of p21 by a p53-independent mechanism. A similar effect was previously reported, but was insufficient to exert any inhibitory effect on cell cycle progression [22]. In addition, overexpression of p53 (Figure 6C and 6D) on rat PC12 cells, and activation of endogenous p53 by etoposide treatment also decreased SALL2 protein levels on PC12 and human HCT116 cells (Figure 6E and 6F).

**Figure 7:**
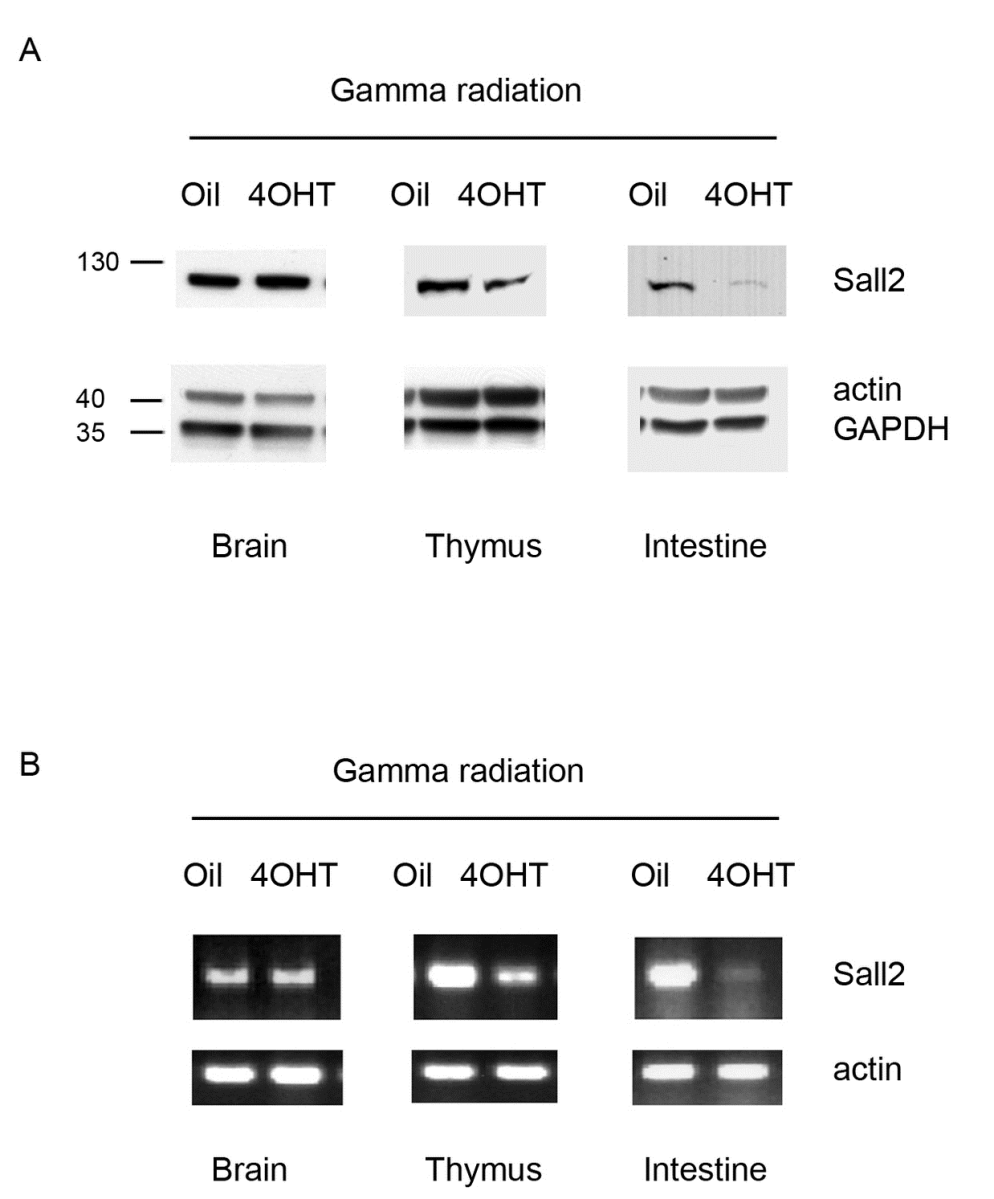
Activation of p53 by genotoxic insults decreases Sall2 levels *in vivo*. p53 ^ER/ER^ mice were treated with 4OH Tamoxifen or vehicle and then exposed to gamma radiation as described in Material and Methods. **A**. Western blot analysis of Sall2 protein levels using total lysates obtained from sections of brain, thymus and small intestine from the p53 ^ER/ER^ mice. Actin and GAPDH show equal loading. Molecular weight markers are indicated on the left side. **B**. RT-PCR analysis of Sall2 gene on total RNA isolated from sections of brain, thymus and small intestine and brain from p53 ^ER/ER^ mice. Actin was used as normalizing gene. All tissues tested were from four individual mouse /condition. Representative tissues lysates are shown.

P53 has a crucial role in protecting cells from pathological outcomes due to genotoxic insults, such as γ-irradiation, by triggering growth arrest or apoptosis in radiosensitive tissues such as intestinal epithelium, spleen, bone marrow, thymus, tongue, testis and hair follicles [43–46]. As doxorubicin and etoposide are genotoxic agents, and inhibited the expression of SALL2 *in vitro*, we evaluated the dependence of acute genotoxic responses for the effect of p53 on SALL2 levels *in vivo*. We treated 8-week-old p53 ^ER/ER^ mice with systemic 4-hydroxytamoxifen over 4 hours to establish wild-type p53 status as previously reported [22,47]. Control mice were treated with equivalent doses of peanut oil carrier alone. We then exposed mice to 5.0 Gy of whole body γ-radiation, collected radiosensitive tissues (spleen, thymus and intestinal epithelium) 4 hours after the exposure and tested for SALL2 expression. Confirming all our previous studies, γ-irradiation decreased SALL2 mRNA and protein levels in radiosensitive tissues from mice pretreated with 4-hydroxytamoxifen, but not in resistant tissues such as the brain (Figure 7A and 7B). All these results demonstrated that there is a p53-dependent inhibition of SALL2 expression under genotoxic cellular stress.

## Discussion

Several evidences indicate that the SALL2 transcription factor behaves as a tumor suppressor gene in cancer. Not in complete agreement with this hypothesis is that SALL2 is found upregulated in various human cancers, and that Sall2 knockout mouse models that currently exits do not show spontaneous tumor formation. In order to understand how a tumor suppressor gene could be upregulated in cancer, we investigated transcriptional regulation of SALL2 and identified it as a novel downstream target of the p53 tumor suppressor protein.

By bioinformatic analysis of the SALL2 gene, we identified several putative p53 half sites in the human SALL2 promoters. The p53 half site located at position -147 (proximal region) of the P2 Sall2 promoter, C **CT** GCCC is conserved in mouse and human promoters and contains the CWWG motif, a key factor that determines whether p53 transcriptionally activates o represses a target gene [29]. Recent studies indicate that a p53 response element (RE) with a dinucleotide core of either AT, AA or TT is activating, whereas a dinucleotide core of CG, GC, TG, CC, GC or CA is repressing. The remaining dinucleotides possibilities (TA, AC, AG, CT, GT, TC or GA) can be activating or repressing depending on the background of the p53RE half-site location [29]. In the case of the SALL2 promoter, the conserved CWWG motif contains a CT sequence defining that the p53RE site could be activating or repressing. Our pGL3-luciferase reporter assays (Figures 2 and 3) suggested that the p53 RE site found has a repressor activity on the regulation of the SALL2 promoter. In addition, the presence of a functional p53RE site is further supported by ChIP assays that confirmed *in vivo* binding of p53 to the SALL2 promoter under doxorubicin treatment (Figure 5).

Supporting the p53-dependent repression of the SALL2 gene, we showed that the activity of the SALL2 P2 promoter is dramatically decreased by either overexpression of wild type p53 in 293T (p53 inactive), H1299 (p53-null), HCT116 (p53-null) and p53-null MEFs, or by activation of endogenous p53 with doxorubicin in HCT116 (p53 wild type) and MEFs (p53 competent) cells. While the mechanism of transcriptional induction by p53 is well-characterized, p53-dependent transcriptional repression is less understood [48]. P53 can repress genes by direct interaction with target gene promoters or bound factors, or indirectly by the action of other p53 target genes [48,49]. In any case, the mechanisms of transcriptional repression by p53 require its intact DNA binding domain. Our data showed that p53 represses SALL2 transcription and that p53 binding to the SALL2 promoter is relevant in this effect. We tested the effect of three well-characterized p53 hot spot mutants, R175H, R249S, and R248W, on P2 activity, but none of them were able to affect it. Loss of function mutations in p53 destabilizes thermodynamically the DNA binding domain, thereby not only reducing the expression of genes that are transactivated by p53 but also derepressing genes that are normally suppressed by p53. An example of this phenomenon is the derepression of the CD44 receptor in the absence of functional p53 [50,51]. Thus, the lack of SALL2 regulation by the R175H, R249S, and R248W mutants suggests that p53 specific binding to DNA is necessary for the regulation of SALL2. By EMSA, we demonstrated that p53 specifically binds to the proximal region (-147 position) of the SALL2 P2 promoter (Figure 4). In addition, this interaction of p53 with the SALL2 P2 promoter was confirmed in vivo by the Chip assay (Figure 5). It has been reported that p53 can bind directly to its response element and recruit corepressors. One such example involves the recruitment of HDAC1 to specific promoters (such as map4, p21, stathmin, HSP90-beta, myc, or nanog) via a p53-dependent interaction with mSIN3A or Zbtb4 [52]. However, direct transcriptional repression by p53 could also involve binding of p53 to an element which overlaps binding sites with coactivator molecules, such as occurs in the humans polycystic kidney disease-1, and AP-endonuclease promoters [53,54]. The p53 half site we identified on the SALL2 promoter locates between two overlapping AP4 binding sites and in the vicinity of a putative SP1 site ( [20] and Figure 1B). Since AP4 has been recently described as a positive regulator of SALL2 transcription under serum starvation, a possible explanation for SALL2 repression might involve binding of p53 interfering with the binding of AP4 or SP1. However, the underlying mechanism of the repression of Sall2 by p53 requires further investigation.

To directly define cellular conditions that trigger a p53-dependent repression of SALL2, we have used a switchable knock-in mouse model, and cells derived from it. In this model, endogenous p53 can be reversible switched between functional and nonfunctional states by modifying endogenous p53 to encode the 4-hydroxytamoxifen-dependent p53ERTAM protein [22]. Expression of p53ERTAM is controlled by the same transcriptional regulatory sequences that normally regulate p53 expression in each tissue *in vivo*, but the expressed p53ERTAM protein is only functionally competent in the presence of 4-hydroxytamoxifen. We demonstrated that the effect of p53 on SALL2 was only observed under p53 activation, as basal levels of SALL2 did not differ between MEFs treated or not with 4-hydroxytamoxifen. Importantly, we confirmed the physiological relevance of these observations in tissues from p53^ER/ER^ mice exposed to gamma radiation. We found that in radiosensitive tissues, such as thymus and intestinal epithelium, SALL2 levels decrease. However, activation of p53 by gamma radiation in low radiosensitive tissue, such as brain, did not affect SALL2 levels suggesting that the regulatory effect of p53 on SALL2 is tissue-specific. The dependence on p53 activation for the effect on SALL2 levels was also observed in PC12 rat cells and human colon cancer HCT116 cells exposed to etoposide (Figure 5E and 5F). In contrast to our observation of the effect of etoposide on SALL2 protein levels, a modest increase on SALL2 mRNA levels in response to a longer exposure and much higher concentration of etoposide has been described in HOSE cells [55]. Although it is unclear the reason for this discrepancy, in agreement with our results, data from ArrayExpress (http://www.ebi.ac.uk/arrayexpress/) show a significant decrease on Sall2 expression under doxorubicin and etoposide treatment in rat hepatocytes. In addition, data from Geo Expression Omnibus (www.ncbi.nlm.nih.gov/geo) show a decrease on Sall2 levels under doxorubicin treatment in MCF7 (**GSE24065**) and mouse embryonic stem cells (**GSE26360**) (Figure S4).

Gene repression by p53 contributes to its tumor suppressive activity; many genes repressed by p53 are involved in cell cycle progression thereby contributing to p53-induced cell cycle arrest [56], and in cell survival under hypoxia thereby contributing to p53-induced apoptosis [57]. These evidences may indicate that SALL2 has functions opposite to those of p53. However, previous studies indicate that SALL2 acts as a tumor suppressor and can regulate p21 ^WAF^ expression independently of p53 [8]. In addition, recent studies indicate that tumor susceptible mice (p53^-/-^) crossed with Sall2-deficient mice (Sall2 ^-/-^ or Sall2 ^-/+^/p53 ^-/-^ mice) exhibited significantly accelerated tumorigenesis and mortality rate compared to Sall2 ^+/+^/ p53^-/-^ mice suggesting a synergy between p53 and SALL2 [58]. Thus, rather than SALL2 opposing p53, all these studies indicate that SALL2 might have p53-dependent and -independent functions that contribute to an outcome similar to that induced by P53. Under these considerations, it is in principle surprising that a tumor suppressor protein represses another tumor suppressor. However, a negative regulation of p53 on other tumor suppressors such as PTEN, ARF, and HIPK2 has been previously described [59–61]. As our data, together with the microarray data, strongly support a negative regulation of SALL2 by p53 under genotoxic stress, the opposing relationship between SALL2 and p53 seems to be only context-dependent. In this regard, it is possible that Sall2 acts as an oncogene in some settings. Some examples of tumor suppressors or oncogenes with dual functions exit, including Myc [62], Runx3 [63], Notch [64], p21 [65] just to mention a few. Sall2 overexpression has been reported in some tumors such as Wilm’s tumor [10], synovial sarcomas [11], squamous cell carcinoma (SCC) of the tongue, and Testicular Germ Cell tumor (TGC) [14]. Although the status of Sall2 in these tumors is unknown, it is possible that its deregulation results in adverse cell behaviors.

Understanding SALL2 regulation should provide insights about its deregulation in cancer. While decreased SALL2 transcripts were found in human acute myeloid leukemias (AMLs) and in ovarian carcinomas [21,58], as mentioned above, SALL2 is found upregulated in Synovial Sarcoma, Wilm’s tumor, oral and testicular cancer [11,12,14]. Within the mechanisms that could explain an upregulation of SALL2 expression in some cancers, it was recently shown that, similar to the binding of the large T protein of mouse polyomavirus to SALL2, the human papillomavirus type 16 E6 protein also interacts with SALL2 thereby stabilizing and increasing levels of a non-functional SALL2 protein [66]. In addition, WT1 was shown to repress SALL2 promoter activity [19], which may provide an explanation for the upregulation of SALL2 in Wilm’s tumors resulting from WT1 inactivation. Here we showed that SALL2 expression is repressed during genotoxic stress in a p53-dependent manner. Interesting, the p53 mutants that were unable to affect SALL2 expression are frequently found in the tumors of cancer patients. Under genotoxic stress, wild type p53 can prevent tumor formation by inducing cell cycle arrest or apoptosis depending on extend of DNA damage. Thus, mutational inactivation of p53 predisposes cells for transformation, and renders cancer cells more resistant to therapies due to lack of p53-mediated apoptosis. Considering that wild type p53, but none of the p53 mutants tested can affect SALL2 levels, we speculate that cancer patients could have low or high levels of SALL2 depending on the p53 status and treatment. It would be interesting to establish whether a correlation between p53 status and SALL2 expression exists in tumors. Still, understanding how upregulation of SALL2 in Wilm’s tumors and other cancers affects tumor biology awaits further studies. In summary, our study highlights a relationship between SALL2 and p53 during genotoxic stress. We predict that future studies on the functional relationship between SALL2 and p53 will provide new avenues through our understanding of normal SALL2 function as well as its role during disease.

## Supporting Information

Figure S1 Putative p53 response elements in the SALL2 gene. The human SALL2 gene (KIAA0360, Hsal2), reference number NM_005407 was analyzed by p53Fam Tag Database (http://p53famtag.ba.itb.cnr.it/) for the presence of putative p53 response elements (p53RE). **A**. Schematic representation of SALL2 gene and the positions of putative p53RE (ovals) identified. The location of exon 1, 1A and 2 are presented as black rectangles. **B**. Table specifies start of p53RE in the SALL2 gene, position from exon 1 and exon 1A, size, direction of stand, localization and sequences of the p53REs.
(DOCX)

Figure S2 Protein-DNA binding analyses for p53-containing nuclear extracts. **A**. Nuclear extracts from HCT116 p53-null cells, transfected or non-transfected with a vector coding for wild-type p53, were analyzed by Western blot using the p53 antibody DO1. GLB = Gel loading buffer. The “x” indicates a non-specific band also detected in the non-transfected cells. **B**. EMSA assay comparing the nuclear extracts described in “A”. The analysis was carried out with a labeled double-stranded oligonucleotide containing the consensus p53 binding sequence (Table S2, named here Cp53). The presence of nuclear extract and PAb421antibody in the binding reactions is depicted at the top of the figure. The migration of the free probe and the DNA/p53/PAb421 complex (arrow) is indicated at the right side of the figure, as well as migration of non-specific complexes generated in the presence of nuclear extracts (X). The nuclear extract incorporated in this assay was obtained from HCT116 p53-null cells transfected with a vector coding for wild-type p53.
(DOC)

Figure S3 The EMSA complex generated with the +282 probe does not correspond to p53 binding. EMSA assay testing the +282 double-stranded oligonucleotide probe, which spans an intronic region of the SALL2 gene (See text for details). The assay used a nuclear extract obtained from HCT116 p53 +/+ cells and includes a competition analysis, consisting in incubation with a 25x molar excess of the unlabeled oligonucleotides Cp53 (double-stranded oligonucleotide containing the consensus p53 binding site) and the +282 oligonucleotide itself. The presence of nuclear extract, PAb421 antibody and unlabeled oligonucleotides in the binding reactions is indicated at the top of the figure. The # symbol indicates the migration of the EMSA complex generated with the use of this probe. Migration of the free probe is also indicated at the right side of the figure.
(DOC)

Figure S4 SALL2 mRNA levels decrease in response to genotoxic agents. Microarray data obtained from Geo Expression Omnibus (www.ncbi.nlm.nih.gov/geo) **A. GSE24065 array experiment**: “Crossroads of the p53, ER, NFkβ stress response networks in MCF7 cells”. Graphs were obtained with GEO2R and show levels of SALL2 and CDKN1A in response to doxorubicin treatment. **B**. **GSE26360 array experiment**: “Genome-wide analysis revealed a crosstalk between p53 and the pluripotent gene networks in mouse embryonic stem cells” (Li M, He Y, Dubois W, Wu X et al*. Mol Cell* 2012 Apr 13; 46(1): 30-42. PubMed: 22387025). Graphs were obtained with GEO2R and show levels of SALL2 and CDKN1A in response to doxorubicin treatment. **C**. Table obtained from **experiment E-MTAB-797** in ArrayExpress (http://www.ebi.ac.uk/arrayexpress/). Transcription profiling of rat hepatocytes treated with approximately 130 chemicals in vitro (3140 assays). Data show downregulation of SALL2 under doxorubicin (0.08 µM and 0.4 µM, p value: 0.001), and etoposide (70 µM, p value: 0.026) treatments.
(DOCX)

Table S1 Primers used for cloning of Sall2 promoter constructs and for PCR on ChIP experiments. (DOCX)

Table S2 Sequences of oligonucleotides used in EMSA (core sections of p53 binding sequences are underlined). (DOCX)
