# Correction: Wild Type p53 Transcriptionally Represses the SALL2 Transcription Factor under Genotoxic Stress

**DOI:** 10.1371/journal.pone.0104307

**Published:** 2014-08-07

**Authors:** The PLOS ONE Staff

Dr David B. Donner was incorrectly listed as fifth author of this article. The editors have followed up with the authors' institutions, the Universidad de Concepción and the University of California San Francisco, in relation to the contributions to this work.

In line with the recommendation by the University of California San Francisco, where the work described in this article was initiated, Dr Donner should be listed as co-corresponding author and placed before Dr Pincheira's name. The author list should be revised to read as follows:

Carlos Farkas, Carla P. Martins, David Escobar, Matias I. Hepp, Ariel F. Castro, Gerard Evan, José L. Gutiérrez, Robert Warren, David B. Donner^☯*^, Roxana Pincheira^☯*^

^*^Corresponding Authors: donnerd@surgery.ucsf.edu (DBD); ropincheira@udec.cl (RP)

In addition, the Funding statement should be revised to acknowledge support to Dr Warren and Dr Donner, the Funding statement should read as below:

This work was supported by Academic Senate Individual Investigator Grant from University of California, San Francisco to RP, DIUC grant from Universidad de Concepcion-Chile, and Regular Fondecyt Grant 1110821-Chile to RP. CF was supported by the Regular Fondecyt Grant 1110821-Chile to RP. DE was supported by a PhD fellowship from CONICYT-Chile. RW was supported by a grant by the Edmund Wattis Littlefield Foundation, DD was supported by the National Institutes of Health (NIH) Grant CA67891, a grant from the Cancer Research Coordinating Committee of the University of California and a grant from the Lehman Brothers Research Foundation.The funders had no role in study design, data collection and analysis, decision to publish, or preparation of the manuscript.
