# Supplementary material for: Wild Type p53 Transcriptionally Represses the SALL2 Transcription Factor under Genotoxic Stress
Source: PLoS One. 2013 Sep 6;8(9):e73817. doi: 10.1371/journal.pone.0073817 (PMC3765348; doi:10.1371/journal.pone.0073817)
Supplement: Figure S1 — The human SALL2 gene (KIAA0360, Hsal2), reference number NM_005407 was analyzed by p53Fam Tag Database (http://p53famtag.ba.itb.cnr.it/) for the presence of putative p53 response elements (p53RE). A. Schematic representation of SALL2 gene and the positions of putative p53RE (ovals) identified. The location of exon 1, 1A and 2 are presented as black rectangles. B. Table specifies start of p53RE in the SALL2 gene, position from exon 1 and exon 1A, size, direction of stand, localization and sequences of the p53REs. (DOCX) [file pone.0073817.s001.docx]

**A**

-3000 -1132 -4700 -4300

12kb intron 0.4kb intron

E1 E1A E2

**B**

| Start | Position from exon 1 or 1A | Size | Strand | Localization | Sequence |
| --- | --- | --- | --- | --- | --- |
| 21068404 | -4300 | 34 | -1 | intron | ATACAAAATTAGCCGGGCTTGGTGGCACATGCCT |
| 21068725 | -4700 | 38 | -1 | intron | AAAATATTTTAATTAGCTGGGCATGATTGTGCATGCCT |
| 21075994 | -1132 | 34 | -1 | promoter | AGACGGGGTTTCAACATGTTGGTCAGGCTGGTCT |
| 21078327 | -3000 | 34 | -1 | promoter | ATACAAAATCAGCCAGGCATGGTGGCACATGCCT |

**Supplementary Figure S1: Putative p53 response elements in the *SALL2* gene**.
