# Supplementary material for: Wild Type p53 Transcriptionally Represses the SALL2 Transcription Factor under Genotoxic Stress
Source: PLoS One. 2013 Sep 6;8(9):e73817. doi: 10.1371/journal.pone.0073817 (PMC3765348; doi:10.1371/journal.pone.0073817)
Supplement: Figure S2 — A. Nuclear extracts from HCT116 p53-null cells, transfected or non-transfected with a vector coding for wild-type p53, were analyzed by Western blot using the p53 antibody DO1. GLB = Gel loading buffer. The “x” indicates a non-specific band also detected in the non-transfected cells. B. EMSA assay comparing the nuclear extracts described in “A”. The analysis was carried out with a labeled double-stranded oligonucleotide containing the consensus p53 binding sequence (Table S2, named here Cp53). The presence of nuclear extract and PAb421antibody in the binding reactions is depicted at the top of the figure. The migration of the free probe and the DNA/p53/PAb421 complex (arrow) is indicated at the right side of the figure, as well as migration of non-specific complexes generated in the presence of nuclear extracts (X). The nuclear extract incorporated in this assay was obtained from HCT116 p53-null cells transfected with a vector coding for wild-type p53. (DOC) [file pone.0073817.s002.doc]

**Supplementary Figure S2.** Protein-DNA binding analyses for p53-containing nuclear extracts.
