# Supplementary figures and images for: Wild Type p53 Transcriptionally Represses the SALL2 Transcription Factor under Genotoxic Stress
Source: PLoS One. 2013 Sep 6;8(9):e73817. doi: 10.1371/journal.pone.0073817 (PMC3765348; doi:10.1371/journal.pone.0073817)

**
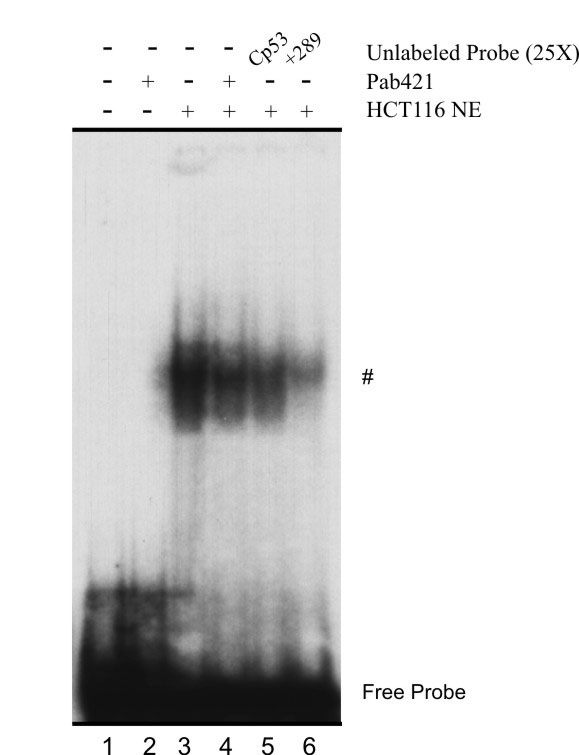
**

**Supplementary Figure S3. Complex generated with the +282 probe does not correspond to p53 binding**

Supplement: Figure S3 — EMSA assay testing the +282 double-stranded oligonucleotide probe, which spans an intronic region of the SALL2 gene (See text for details). The assay used a nuclear extract obtained from HCT116 p53 +/+ cells and includes a competition analysis, consisting in incubation with a 25x molar excess of the unlabeled oligonucleotides Cp53 (double-stranded oligonucleotide containing the consensus p53 binding site) and the +282 oligonucleotide itself. The presence of nuclear extract, PAb421 antibody and unlabeled oligonucleotides in the binding reactions is indicated at the top of the figure. The # symbol indicates the migration of the EMSA complex generated with the use of this probe. Migration of the free probe is also indicated at the right side of the figure. (DOC) [file pone.0073817.s003.doc]
