# Supplementary material for: Wild Type p53 Transcriptionally Represses the SALL2 Transcription Factor under Genotoxic Stress
Source: PLoS One. 2013 Sep 6;8(9):e73817. doi: 10.1371/journal.pone.0073817 (PMC3765348; doi:10.1371/journal.pone.0073817)
Supplement: Figure S4 — Microarray data obtained from Geo Expression Omnibus (www.ncbi.nlm.nih.gov/geo) A. GSE24065 array experiment: “Crossroads of the p53, ER, NFkβ stress response networks in MCF7 cells”. Graphs were obtained with GEO2R and show levels of SALL2 and CDKN1A in response to doxorubicin treatment. B. GSE26360 array experiment: “Genome-wide analysis revealed a crosstalk between p53 and the pluripotent gene networks in mouse embryonic stem cells” (Li M, He Y, Dubois W, Wu X et al. Mol Cell 2012 Apr 13; 46(1): 30-42. PubMed: 22387025). Graphs were obtained with GEO2R and show levels of SALL2 and CDKN1A in response to doxorubicin treatment. C. Table obtained from experiment E-MTAB-797 in ArrayExpress (http://www.ebi.ac.uk/arrayexpress/). Transcription profiling of rat hepatocytes treated with approximately 130 chemicals in vitro (3140 assays). Data show downregulation of SALL2 under doxorubicin (0.08 µM and 0.4 µM, p value: 0.001), and etoposide (70 µM, p value: 0.026) treatments. (DOCX) [file pone.0073817.s004.docx]

**A.**

*
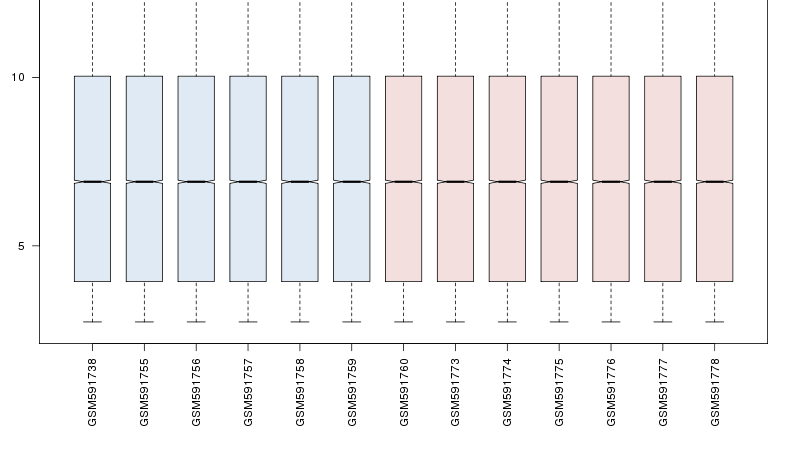

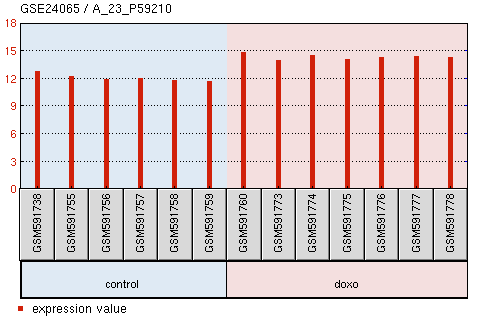

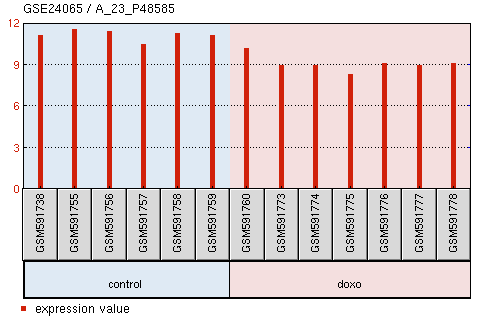
SALL2 CDKN1A* Data deviation

**B.**

*
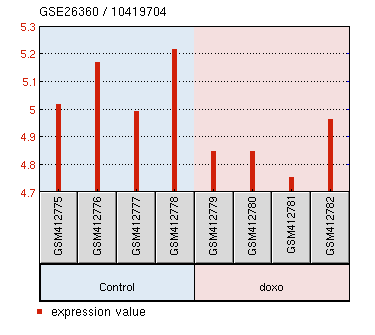

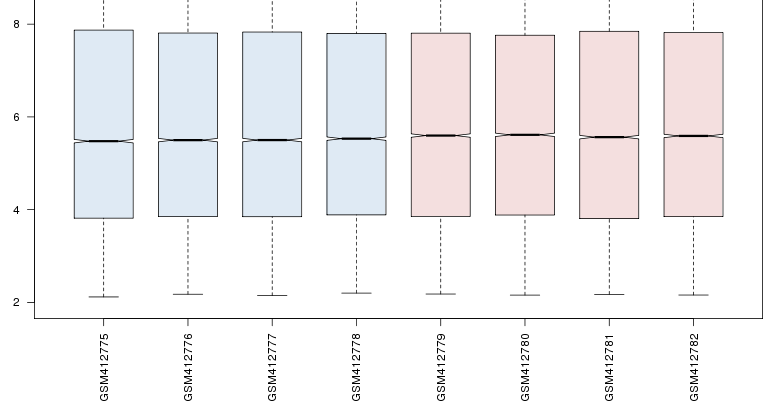

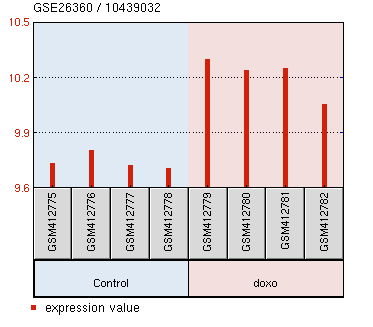
SALL2 CDKN1A* Data deviation

**C.**

| **Treatment** | **Factor Value** | **UP/DOWN** | **T-statistic** | **P-value** |
| --- | --- | --- | --- | --- |
| Doxorubicin | 0.08 μM | DOWN | -3.8 | 0.001 |
| Doxorubicin | 0.4 μM | DOWN | -3.9 | 0.001 |
| Doxorubicin | 2 μM | NONDE | -1.4 | 0.287 |
| Etoposide | 14 μM | NONDE | -1.3 | 0.355 |
| Etoposide | 35 μM | NONDE | -1.3 | 0.355 |
| Etoposide | 70 μM | DOWN | -2.8 | 0.026 |

**Supplementary Figure S4.** **SALL2 mRNA levels decrease in response to genotoxic agents.**
