# Supplementary material for: Wild Type p53 Transcriptionally Represses the SALL2 Transcription Factor under Genotoxic Stress
Source: PLoS One. 2013 Sep 6;8(9):e73817. doi: 10.1371/journal.pone.0073817 (PMC3765348; doi:10.1371/journal.pone.0073817)
Supplement: Table S1 — (DOCX) [file pone.0073817.s005.docx]

**Supplementary Table S1.**

| **Amplicon /Use** | **Sense 5’🡪 3’** | **Antisense 5’ 🡪 3’** |
| --- | --- | --- |
| 1.2 Kb Sall2 promoter/cloning | GGTCACTCGAGAATGACTTCCTGGGA | AACCAAAGCTTCTAATTAACAAGGAG |
| 1.1 Kb upstream Sall2 promoter/cloning | GATCAAATGGTACCCCATCATGCCCAGCTAATTT | GATCAAATCTCGAGTCAGGACTCTCTGCGGAACACT |
| Proximal E1A/ChIP | CTCTCCCCGCTTTCTCACT | CAGACTGCGGAGATGGAGAT |
| Distal E1A/ChIP | CCAGTAGCTGGGATTAAAGGTG | AGATCACGAGGTCAGAAGTTCC |
| Intron Sall2/ChIP | CCGTGAACTCGCTTGTGG | ATCCGTGTGGACAGGAGACA |
| p21 positive control /ChIP | GTGGCTCTGATTGGCTTTCTG | CTGAAAACAGGCAGCCCAAG |
