# Supplementary material for: Wild Type p53 Transcriptionally Represses the SALL2 Transcription Factor under Genotoxic Stress
Source: PLoS One. 2013 Sep 6;8(9):e73817. doi: 10.1371/journal.pone.0073817 (PMC3765348; doi:10.1371/journal.pone.0073817)
Supplement: Table S2 — (DOCX) [file pone.0073817.s006.docx]

**Supplementary Table S2**

| Sequence | Name | Location of p53 site on Sall2 gene |
| --- | --- | --- |
| ATTAAAGGTGTACCAT**CATG**CCCAGCT | -1879 S | -1879 |
| AGCTGGGCATGATGGTACACCTTTAAT | -1879 A |  |
| GCTGCTC**CCTG**CCCCCGC**CGCTG**CCGATCT | -147 S | -147 |
| AGATCGGCAGCGGCGGGGGCAGGGAGCAGC | -147 A |  |
| GCTGCTC**tCTa**CCCCCGCCGCTGCCGATCT | -147 mut S | -147 mutant |
| AGATCGGCAGCGGCGGGGGtAGaGAGCAGC | -147 mut A |  |
| CCCACCCC**CCTG**CCC**CATG**CCGGGTTG | +282 S | +282 |
| CAACCCGGCATGGGGCAGGGGGGTGGG | +282 A |  |
| TACAGAA**CATG**TCTAAG**CATG**CTGGGGA | Consensus p53 S |  |
| TCCCCAGCATGCTTAGACATGTTCTGTA | Consensus p53 A |  |
